# Urinary NGAL as a Diagnostic and Prognostic Marker for Acute Kidney Injury in Cirrhosis: A Prospective Study

**DOI:** 10.14309/ctg.0000000000000359

**Published:** 2021-05-11

**Authors:** Andrew S. Allegretti, Xavier Vela Parada, Paul Endres, Sophia Zhao, Scott Krinsky, Shelsea A. St. Hillien, Sahir Kalim, Sagar U. Nigwekar, James G. Flood, Andrea Nixon, Douglas A. Simonetto, Luis A. Juncos, Nithin Karakala, Hani M. Wadei, Kevin R. Regner, Justin M. Belcher, Mitra K. Nadim, Guadalupe Garcia-Tsao, Juan Carlos Q. Velez, Samir M. Parikh, Raymond T. Chung

**Affiliations:** 1 Division of Nephrology, Department of Medicine, Massachusetts General Hospital, Boston, Massachusetts, USA;; 2 Department of Pathology, Massachusetts General Hospital, Boston, Massachusetts, USA;; 3 Division of Gastroenterology and Hepatology, Mayo Clinic, Rochester, Minnesota, USA;; 4 Department of Medicine, University of Arkansas for Medical Sciences, Central Arkansas Veterans Healthcare System, Little Rock, Arkansas, USA;; 5 Department of Transplantation, Mayo Clinic, Jacksonville, Florida, USA;; 6 Division of Nephrology, Medical College of Wisconsin, Milwaukee, Wisconsin, USA;; 7 Section of Nephrology, Yale University School of Medicine, New Haven, Connecticut, USA and Section of Nephrology, VA-Connecticut Healthcare System, West Haven, Connecticut, USA;; 8 Division of Nephrology and Hypertension, Keck School of Medicine, University of Southern California, Los Angeles, California, USA;; 9 Section of Digestive Diseases, VA-Connecticut Healthcare System, West Haven, Connecticut, USA;; 10 Department of Nephrology, Ochsner Health System, Baton Rouge, Louisiana, USA;; 11 Division of Nephrology, Department of Medicine, Beth Israel Deaconess Medical Center, Boston, Massachusetts, USA;; 12 Liver Center and Gastrointestinal Division, Department of Medicine, Massachusetts General Hospital, Boston, Massachusetts, USA.

## Abstract

Urinary neutrophil gelatinase-associated lipocalin (NGAL) has shown promise in differentiating acute tubular necrosis (ATN) from other types of acute kidney injuries (AKIs) in cirrhosis, particularly hepatorenal syndrome (HRS). However, NGAL is not currently available in clinical practice in North America.

**METHODS:**

Urinary NGAL was measured in a prospective cohort of 213 US hospitalized patients with decompensated cirrhosis (161 with AKI and 52 reference patients without AKI). NGAL was assessed for its ability to discriminate ATN from non-ATN AKI and to predict 90-day outcomes.

**RESULTS:**

Among patients with AKI, 57 (35%) had prerenal AKI, 55 (34%) had HRS, and 49 (30%) had ATN, with a median serum creatinine of 2.0 (interquartile range 1.5, 3.0) mg/dL at enrollment. At an optimal cutpoint of 244 μg/g creatinine, NGAL distinguished ATN (344 [132, 1,429] μg/g creatinine) from prerenal AKI (45 [0, 154] μg/g) or HRS (110 [50, 393] μg/g; *P* < 0.001), with a C statistic of 0.762 (95% confidence interval 0.682, 0.842). By 90 days, 71 of 213 patients (33%) died. Higher median NGAL was associated with death (159 [50, 865] vs 58 [0, 191] μg/g; *P* < 0.001). In adjusted and unadjusted analysis, NGAL significantly predicted 90-day transplant-free survival (*P* < 0.05 for all Cox models) and outperformed Model for End-Stage Liver Disease score by C statistic (0.697 vs 0.686; *P* = 0.04), net reclassification index (37%; *P* = 0.008), and integrated discrimination increment (2.7%; *P* = 0.02).

**DISCUSSION:**

NGAL differentiates the type of AKI in cirrhosis and may improve prediction of mortality; therefore, it holds potential to affect management of AKI in cirrhosis.

## BACKGROUND

Acute kidney injury (AKI) is a common and highly morbid complication of decompensated cirrhosis (1–3). AKI in cirrhosis is defined and staged by relative changes in serum creatinine (3,4). Higher AKI stage (i.e., more severe injury) is associated with increased 90-day mortality; stage 3 AKI (up to 60% mortality) and dialysis-dependent AKI (>80% mortality) convey the worst short-term prognosis (5–8). Overall, AKI-associated mortality risk in cirrhosis depends on several factors, including severity and cause of AKI, degree of liver failure, and other ongoing complications of liver disease (9–11). Thus, it is critical to diagnose AKI quickly and accurately to allow for early therapeutic intervention, thereby increasing the odds of reversing the dysfunction.

Although current creatinine-based definitions play a key role in the diagnosis and prognosis of AKI in cirrhosis, several limitations remain (12). Importantly, serum creatinine does not inform the type or cause of AKI (13). There are 3 common types of AKIs in cirrhosis: prerenal AKI (which is generally reversible with volume resuscitation and discontinuation of diuretics), hepatorenal syndrome (HRS), and acute tubular necrosis (ATN) (14). Differentiating HRS from ATN is difficult because there is no available objective test to distinguish between the 2, leaving clinicians to make the diagnosis largely on clinical grounds (3,15). Accurate diagnosis has major therapeutic implications because HRS and ATN are treated differently. HRS is managed with vasoconstrictors and volume expansion, whereas ATN often warrants a fluid-restrictive and supportive strategy (3,16,17). As a result, international guidelines have called for the increased study of novel kidney injury biomarkers that hold potential to differentiate between ATN and other causes of AKI in cirrhosis (3).

Recently, urinary tubular injury markers have shown promise in differentiating HRS from ATN in cirrhosis. In particular, urinary neutrophil gelatinase-associated lipocalin (NGAL) has demonstrated the best performance among a group of similar markers (18–20). However, NGAL is not available in clinical practice in North America, and the largest studies of NGAL in cirrhosis are from Europe (21–23). Therefore, we undertook the largest study of North American patients to validate NGAL as a diagnostic and prognostic tool for AKI in cirrhosis.

## METHODS

### Population and setting

Between 2013 and 2019, nonconsecutive adult patients (age 18 years or older) admitted to Massachusetts General Hospital (Boston, MA) with decompensated cirrhosis and AKI (plus a reference group admitted with complications of cirrhosis and no AKI) were enrolled and followed prospectively for 90 days from the date of admission. Patients were screened from new admissions censuses using the electronic record. Patients were excluded if they were pregnant, nursing, previously received renal replacement therapy, or had a previous liver or kidney transplant.

### Definitions

Diagnosis and cause of cirrhosis were determined by treating clinical hepatologists. The International Club of Ascites criteria were used to diagnose AKI (3). In brief, AKI was staged by a relative change in serum creatinine (at least 50% increase from last outpatient serum creatinine or an increase of >0.3 mg/dL in the prior 7 days). The type of AKI was determined by history and clinical parameters, as described in previous studies (14,24): prerenal AKI required resolution of AKI (creatinine improved to 0.3 mg/dL of baseline) with volume administration, ATN required a clinical history consistent with ischemic or nephrotoxic AKI and failure to respond to volume administration (using narrative clinical notes, nephrologists' assessment, and objective urinalysis findings, where available), and HRS was diagnosed by exclusion of other potential causes of AKI using the International Club of Ascites HRS-AKI criteria (3).

### Data collection

All data (outside of NGAL measurements) were taken from the electronic medical record. Model for End-Stage Liver Disease (MELD), MELD-Na, and Chronic Liver Failure Consortium Acute-on-Chronic Liver Failure (CLIF-C ACLF) scores were calculated at the time of hospital admission (25–27). Patients provided serum and urine samples at enrollment, day 5, and day 30 after enrollment (the latter 2 if available). Unless otherwise noted, all laboratory values were recorded at the time closest to enrollment sample collection. Study team members did not intervene in the clinical care. Clinical outcomes, including mortality, transplant status, and resolution/progression of AKI, were recorded at hospital discharge and at 90 days after admission. No patients were lost to follow-up.

### NGAL measurement

Urinary NGAL levels were measured using a turbidimetric immunoassay (Bioporto Diagnostics, Hellerup, Denmark) on a Cobas 502 clinical chemistry analyzer (Roche, Basel, Switzerland). NGAL levels were normalized for urine creatinine and reported in μg/g creatinine. All study samples were processed within 4 hours of collection and stored at −80°C. All NGAL measurements were processed in batch at the end of the study. The coefficient of variance for this assay was <10%. NGAL values were not made available to the clinical care teams or to patients.

### Analysis and outcomes

Among patients with AKI, the primary analysis compared urinary NGAL levels between ATN and non-ATN groups. The optimal cutpoint to diagnose ATN was identified using the Youden index (J) method (28). The primary survival outcome (90-day transplant-free survival) (29) was visualized using a Kaplan-Meier curve and compared across tertiles using a log-rank test. Multiple prespecified Cox regression models were used to evaluate the association between NGAL as a continuous variable and transplant-free survival. Ninety-day mortality (without censoring for liver transplant) was also examined in a sensitivity analysis for the aforementioned Cox models. The results of Cox models were summarized with hazard ratios and Wald asymptotic 95% confidence intervals (CIs). Changes in NGAL levels over time (enrollment, day 5, and day 30) were analyzed longitudinally and compared across different causes of AKI (prerenal AKI, HRS, and ATN).

Continuous variables were presented as median (interquartile range) given nonparametric distribution. The data were compared using a χ^2^ test, a Fisher exact test, or a Wilcoxon rank sum test. The incremental improvement of adding NGAL to the existing predictive models of cirrhosis (MELD score, MELD-Na score, and CLIF-C ACLF score) was measured by change in C statistic, category-free net reclassification index, and integrated discrimination index (30). SAS version 9.4 (Cary, NC) was used for all analyses. Two-tailed *P* value < 0.05 was considered statistically significant.

### Ethics statement

The study was approved by the Mass General Brigham Institutional Review Board. All procedures and practices abide by guidelines set forth by the Declarations of Helsinki and Istanbul. Patients (or their health care designee) provided written informed consent. All authors met International Committee of Medical Journal Editors criteria.

## RESULTS

### Patients and clinical characteristics

In all, 213 patients were included in the final analysis. Among 161 patients with AKI, 57 (35%) had prerenal AKI, 55 (34%) had HRS, and 49 (30%) had ATN. Median serum creatinine was 2.0 (1.5, 3.0) mg/dL at the time of enrollment. For all patients, the median admission MELD score was 23 (17, 30) and the CLIF-C ACLF score was 45 (39, 52). The median time from admission to enrollment was 3 (2, 5) days. After enrollment, 34 of 213 patients (16%) went on to undergo a liver transplantation. Table 1 presents clinical characteristics by the type of AKI.

**Table 1.** Baseline characteristics by the AKI type

	No AKI (n = 52)	Prerenal AKI (n = 57)	HRS (n = 55)	ATN (n = 49)
Age (yr)	56.0 [48.0, 63.5]	58.0 [49.0, 64.0]	58.0 [48.0, 65.0]	59.0 [50.0, 63.0]
Female sex (%)	18 (34.6)	19 (33.3)	16 (29.1)	13 (26.5)
White race (%)	48 (92.3)	50 (87.7)	53 (96.4)	46 (93.9)
Non-Hispanic ethnicity (%)	47 (90.4)	47 (82.5)	50 (90.9)	45 (91.8)
Body mass index (kg/m^2^)	27.5 [24.2, 29.8]	28.4 [24.6, 34.9]	29.0 [24.1, 34.1]	29.2 [23.0, 33.4]
History of diabetes mellitus (%)	11 (21.2)	15 (26.3)	14 (25.5)	14 (28.6)
Primary reason for admission (%)				
Complications of liver disease	36 (69.2)	34 (59.6)	33 (60.0)	24 (49.0)
AKI	0 (0.0)	6 (10.5)	16 (29.1)	11 (22.4)
Infection	9 (17.3)	11 (19.3)	2 (3.6)	8 (16.3)
Other	7 (13.5)	6 (10.5)	4 (7.3)	6 (12.2)
Etiology of cirrhosis (%)				
Hepatitis C	5 (9.6)	11 (19.3)	5 (9.1)	8 (16.3)
Alcohol	18 (34.6)	19 (33.3)	18 (32.7)	14 (28.6)
Nonalcoholic steatohepatitis	4 (7.7)	4 (7.0)	6 (10.9)	7 (14.3)
Multifactorial	14 (26.9)	16 (28.1)	18 (32.7)	9 (18.4)
Other	10 (19.2)	7 (12.3)	7 (12.7)	11 (22.4)
Liver complications before admission (%)				
Ascites requiring previous paracentesis	26 (50.0)	33 (57.9)	39 (70.9)^a^	22 (44.9)
Encephalopathy	14 (26.9)	17 (29.8)	25 (45.5)	10 (20.4)
Gastrointestinal bleeding	19 (36.5)	13 (22.8)	12 (21.8)	9 (18.4)
Spontaneous bacterial peritonitis	7 (13.5)	6 (10.5)	9 (16.4)	4 (8.2)
Hepatocellular carcinoma	3 (5.8)	3 (5.3)	7 (12.7)	5 (10.2)
Transjugular portosystemic shunt	6 (11.5)	4 (7.0)	2 (3.6)	3 (6.1)
Vasoconstrictors during admission (%)				
Midodrine	1 (1.9)	18 (31.6)	41 (74.5)	27 (55.1)
Octreotide	9 (17.3)	26 (45.6)	43 (78.2)	24 (49.0)
Intravenous vasopressor	2 (3.8)	14 (24.6)	18 (32.7)	16 (32.7)
MELD score	18.5 [14.0, 24.0]	19.0 [16.0, 29.0]	27.0 [22.0, 33.0]	27.0 [21.0, 35.0]
CLIF-C ACLF score	39.0 [30.5, 49.0]	43.6 [39.0, 48.5]	47.4 [43.4, 55.0]	47.5 [41.7, 53.7]
Laboratory values				
Sodium (mEq/L)	135 [133, 138]	133 [130, 139]	133 [130, 137]	135 [130, 140]
Creatinine (mg/dL)	0.7 [0.6, 0.9]	1.3 [1.1, 1.8]	2.4 [1.9, 3.0]	2.4 [1.8, 4.1]
White blood count (K/uL)	5.8 [3.5, 10.0]	7.8 [5.5, 11.2]	6.8 [4.8, 10.9]	9.7 [6.3, 13.4]
Hemoglobin (g/dL)	9.7 [8.1, 11.3]	8.8 [8.0, 10.1]	8.7 [7.6, 9.8]	8.7 [7.9, 10.2]
Platelets (K/uL)	69 [46, 116]	93 [62, 148]	77 [58, 107]	96 [58, 167]
Albumin (g/dL)	2.6 [2.4, 2.8]	3.1 [2.6, 3.5]	3.5 [3.1, 3.8]	2.9 [2.6, 3.3]
INR	1.6 [1.4, 2.0]	1.6 [1.3, 2.0]	1.9 [1.6, 2.4]	1.8 [1.5, 2.3]
Total bilirubin (mg/dL)	3.1 [1.7, 8.4]	3.2 [1.5, 8.5]	5.1 [2.1, 15.7]	8.1 [2.5, 24.8]
Aspartate aminotransferase (U/L)	57 [40, 79]	61 [43, 105]	49 [27, 76]	85 [41, 153]
Alanine aminotransferase (U/L)	28 [20, 43]	31 [18, 53]	20 [13, 32]	33 [17, 80]
Alkaline phosphatase (U/L)	112 [84, 158]	102 [76, 156]	95 [79, 132]	135 [99, 191]

Continuous variables given as median (IQR). MELD and CLIF-C ACLF score were taken at hospital admission. All laboratory values were taken at the time of enrollment.

AKI, acute kidney injury; CLIF-C ACLF, CLIF Consortium Organ Failure Acute-on-Chronic Liver Failure score; INR, international normalized ratio; IQR, interquartile range; MELD, Model for End-Stage Liver Disease.

a All patients with HRS had either a history of ascites or ascites on admission to fulfill HRS diagnostic criteria.

### NGAL to discriminate the type of AKI

At enrollment, urinary NGAL levels were lowest in prerenal AKI (45 [0, 154] μg/g), higher in HRS (110 [50, 393] μg/g), and highest in ATN (344 [132, 1,429] μg/g; *P* < 0.001). At an optimal cutpoint of 244 μg/g, NGAL discriminated ATN from non-ATN kidney injury with a C statistic of 0.762 (95% CI 0.682, 0.842), 71% sensitivity, 76% specificity, 56% positive predictive value, and 86% negative predictive value. As reference, patients hospitalized with decompensated cirrhosis without AKI had the lowest urinary NGAL levels (19 [0, 58] μg/g).

### Predictors of 90-day mortality

By 90 days, 71 of 213 patients (33%) died (Table 2). In univariate analysis, older age, higher MELD score, higher CLIF ACLF score, presence of hepatocellular carcinoma, and multiple laboratory abnormalities (higher serum creatinine, higher serum albumin after resuscitation, and higher total bilirubin) were associated with death by 90 days (*P* < 0.05 for all). Similarly, the type of AKI was associated with death, with ATN and HRS having higher mortality rates (49% and 44%, respectively) compared with prerenal AKI (30%) and no AKI (12%; *P* < 0.001).

**Table 2.** Clinical characteristics by vital status at 90 days

	Alive at 90 days (n = 142)	Died by 90 days (n = 71)	*P* value
Age (yr)	55.5 [46.0, 62.0]	62.0 [55.0, 67.0]	<0.001
Female sex (%)	44 (31.0)	22 (31.0)	1.0
White race (%)	131 (92.3)	66 (93.0)	0.85
Non-Hispanic ethnicity (%)	123 (86.6)	66 (93.0)	0.17
Etiology of cirrhosis (%)			0.39
Hepatitis C	22 (15.5)	7 (9.9)	
Alcohol	50 (35.2)	19 (26.8)	
Nonalcoholic steatohepatitis	13 (9.2)	8 (11.3)	
Multifactorial	36 (25.4)	21 (29.6)	
Other	20 (14.1)	15 (21.1)	
Hepatocellular carcinoma (%)	7 (4.9)	11 (15.5)	0.009
Presence of infection (%)	43 (30.3)	25 (35.2)	0.47
MELD score	22.0 [16.5, 29.0]	26.0 [18.0, 32.0]	0.02
CLIF-C ACLF score	43.9 [37.3, 50.9]	47.9 [41.7, 53.5]	0.005
Laboratory values			
Sodium (mEq/L)	134 [130, 138]	135 [131, 140]	0.30
Creatinine (mg/dL)	1.4 [0.8, 2.2]	2.1 [1.5, 3.0]	<0.001
White blood cell count (K/uL)	7.2 [4.7, 10.8]	7.6 [5.4, 13.4]	0.15
Hemoglobin (g/dL)	8.8 [7.8, 10.4]	8.8 [7.9, 10.4]	0.87
Platelets (K/uL)	87 [57, 141]	82 [58, 113]	0.50
Albumin (g/dL)	2.8 [2.5, 3.3]	3.3 [2.6, 3.6]	0.01
INR	1.6 [1.4, 2.1]	1.8 [1.5, 2.1]	0.21
Total bilirubin (mg/dL)	3.7 [1.6, 10.2]	7.0 [2.3, 20.2]	0.02
Aspartate aminotransferase (U/L)	55 [35, 84]	70 [41, 124]	0.05
Alanine aminotransferase (U/L)	26 [16, 44]	29 [17, 59]	0.13
Alkaline phosphatase (U/L)	109 [80, 154]	111 [91, 166]	0.27
AKI stage (%)			<0.001
No AKI	46 (32.4)	6 (8.5)	
Stage 1	32 (22.5)	14 (19.7)	
Stage 2	24 (16.9)	13 (18.3)	
Stage 3	40 (28.2)	38 (53.5)	
Required renal replacement therapy (%)	20 (14.1)	22 (31.0)	0.004
Type of AKI (%)			<0.001
No AKI	46 (88.5)	6 (11.5)	
Prerenal AKI	40 (70.2)	17 (29.8)	
HRS	31 (56.4)	24 (43.6)	
ATN	25 (51.0)	24 (49.0)	
Urinary NGAL (μg/g creatinine)	59 [0, 191]	159 [50, 865]	<0.001

Continuous variables given as median [IQR]. MELD and CLIF-C ACLF score were taken at hospital admission. All laboratory values were taken at the time of enrollment.

AKI, acute kidney injury; ATN, acute tubular necrosis; CLIF-C ACLF, CLIF Consortium Organ Failure Acute-on-Chronic Liver Failure score; HRS, hepatorenal syndrome; INR, International normalized ratio; IQR, interquartile range; MELD, Model for End-Stage Liver Disease; NGAL, neutrophil gelatinase-associated lipocalin.

### NGAL to predict survival and transplant-free survival

**Figure 1.**
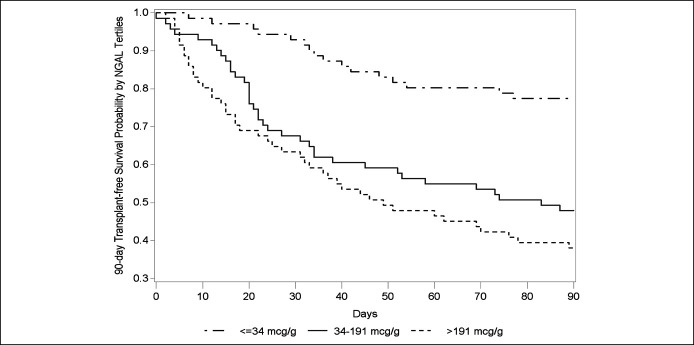
Ninety-day probability of transplant-free survival by urinary NGAL (μg/g creatinine). Patients were divided by NGAL tertile at study enrollment (*P* < 0.001). NGAL, neutrophil gelatinase-associated lipocalin.

Among all 213 patients, those who died had higher median NGAL levels (159 [interquartile range 50, 865] vs 58 [0, 191] μg/g; *P* < 0.001). Kaplan-Meier analysis suggested a graded association between higher NGAL and decreased 90-day transplant-free survival (*P* < 0.001; Figure 1) and 90-day survival (*P* < 0.001; see Supplemental Figure 1, Supplementary Digital Content 1, http://links.lww.com/CTG/A609).

**Figure 2.**
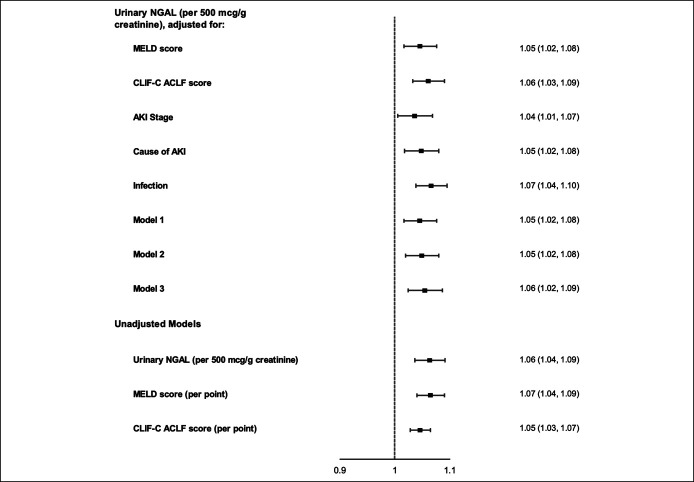
Forest plots of urinary NGAL's hazard ratios for 90-day transplant-free mortality. HRs are presented with 95% CIs. Model 1: adjusted for age and MELD score; model 2: adjusted for age, MELD score, and presence of infection; and model 3: excluding 18 patients with hepatocellular carcinoma. AKI, acute kidney injury; CI, confidence interval; CLIF-C ACLF, Chronic Liver Failure Consortium Acute-on-Chronic Liver Failure score; HR, hazard ratio; MELD, Model for End-Stage Liver Disease.

Multivariable Cox regression models to evaluate NGAL's ability to predict 90-day transplant-free survival are presented in Figure 2. In unadjusted models, higher NGAL was significantly associated with increased risk of transplant or death (*P* < 0.001) with a similar effect estimate to MELD score and CLIF-C ACLF score. All adjusted models showed a stable association between NGAL and the composite outcome of transplant or death (*P* < 0.001 for all). Results were similar in sensitivity analysis when considering 90-day survival as the outcome, without censoring for liver transplantation (*P* < 0.001 for all; see Supplemental Figure 2, Supplementary Digital Content 1, http://links.lww.com/CTG/A609).

### Added predictive value of NGAL to prognostic models in cirrhosis for 90-day outcomes

At an optimal cutpoint of 42 μg/g, urinary NGAL at enrollment predicted 90-day transplant-free mortality with a C statistic of 0.697 (95% CI 0.628, 0.767). In direct comparison, NGAL outperformed MELD score by C statistic (0.697 vs 0.686; *P* = 0.04), category-free net reclassification index (37%; *P* = 0.008), and integrated discrimination increment (2.7%; *P* = 0.02). The results of these analyses were similar when comparing NGAL with the MELD-Na or CLIF-C ACLF score, and when examining 90-day survival (without considering liver transplantation; see Table 3). In a subgroup analysis by the type of AKI, the addition of NGAL improved prediction of 90-day survival in patients with HRS and ATN, but not in the prerenal AKI or no AKI reference groups (see Supplement Figure 3, Supplementary Digital Content 1, http://links.lww.com/CTG/A609).

**Table 3.** Added predictive value of NGAL for 90-day transplant-free survival and 90-day survival to prognostic models in cirrhosis

	NGAL	MELD	MELD + NGAL	*P* value	MELD-Na	MELD-Na + NGAL	*P* value	CLIF-C ACLF	CLIF-C ACLF + NGAL	*P* value
Transplant-free survival										
C statistic	0.697	0.686	0.713	0.04	0.683	0.708	0.06	0.663	0.697	0.04
Category-free NRI			0.368	0.008		0.349	0.01		0.375	0.007
IDI			0.027	0.02		0.029	0.02		0.036	0.007
Overall survival										
C statistic	0.688	0.596	0.662	0.005	0.581	0.653	0.004	0.620	0.675	0.02
Category-free NRI			0.360	0.01		0.388	0.008		0.364	0.01
IDI			0.052	0.007		0.055	0.006		0.056	0.006

ACLF, acute-on-chronic liver failure; CLIF-C ACLF, Chronic Liver Failure Consortium Acute-on-Chronic Liver Failure score; IDI, integrated discrimination increment; MELD, Model for End-Stage Liver Disease; NGAL, neutrophil gelatinase-associated lipocalin; NRI, net reclassification index.

**Figure 3.**
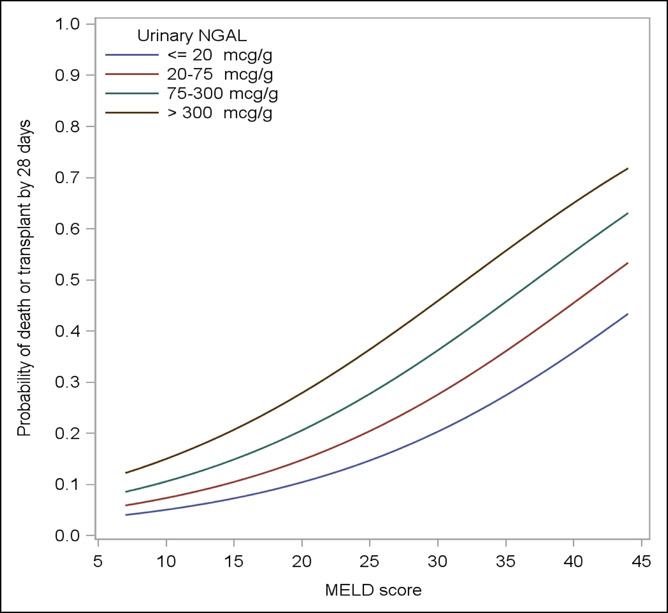
Relationship between MELD score and 28-day transplant-free mortality, adjusted for age and presence of infection, categorized by quartiles of urinary NGAL (μg/g creatinine). MELD, Model For End-Stage Liver Disease; NGAL, neutrophil gelatinase-associated lipocalin.

The added value for NGAL to MELD score is further visualized in Figure 3. Among all patients, the addition of NGAL (in quartiles) refines discrimination of 28-day mortality at a given MELD score, after adjusting for age and presence of infection. For example, at a MELD score of 30, a subject with an NGAL level <20 μg/g (lowest quartile) would have an expected 28-day mortality rate of 20%, whereas an identical subject with an NGAL level >300 μg/g (highest quartile) would have an expected 28-day mortality of 46%.

### NGAL and other outcomes

Higher AKI stage was associated with death by 90 days (12% for no AKI, 30% for stage 1 AKI, 35% for stage 2 AKI, and 49% for stage 3 AKI; *P* < 0.001). NGAL expression increased across AKI stages (53 [0, 154] for stage 1 AKI, 81 [33, 280] for stage 2 AKI, and 294 [82, 1,051] for stage 3 AKI; *P* < 0.001), irrespective of the cause/type of AKI. During admission, 42 of 213 patients (20%) required renal replacement therapy. Higher NGAL at enrollment was associated with increased risk of requiring renal replacement therapy (403 [102, 1,429] vs 58 [16, 190] μg/g; *P* < 0.001). Ninety-day mortality was higher among those who required renal replacement therapy (52% vs 29%; *P* = 0.004). By hospital discharge, 61 of 161 patients (38%) had resolution of AKI (creatinine improved to ≤0.3 mg/dL of baseline) and 34 of 161 patients (21%) had progression of AKI (increase in AKI stage or presenting with stage 3 AKI and later requiring renal replacement therapy). There was a statistically significant difference in enrollment NGAL among those with AKI resolution, those with stable AKI (neither resolution nor progression of AKI), and those with AKI progression (81 [29, 267] vs 111 [45, 330] vs 363 [55, 1,424] μg/g, respectively; *P* = 0.02).

**Figure 4.**
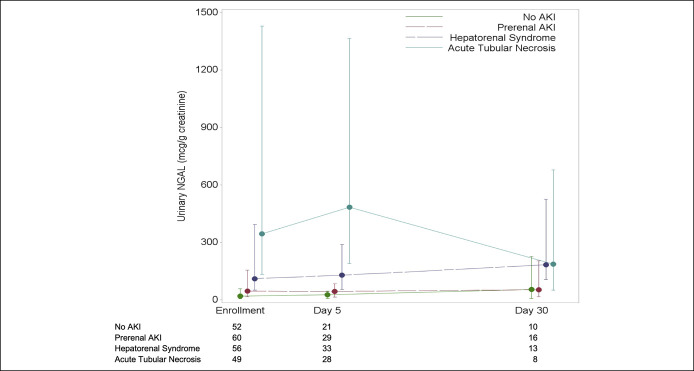
Time course of median urinary NGAL (μg/g creatinine) at study enrollment, day 5, and day 30, classified by the type of AKI. AKI, acute kidney injury; NGAL, neutrophil gelatinase-associated lipocalin.

Figure 4 demonstrates changes in NGAL levels over time by the type of AKI. All 213 patients had a measurement at enrollment; 110 of 213 (52%) had a day 5 sample; and 45 of 213 (21%) had a day 30 sample collected. Patients with ATN had the highest levels on enrollment and at day 5 (*P* < 0.001 for both), although this signal was not present at day 30 (*P* = 0.09). Patients with HRS had increases in NGAL from enrollment to day 5 and from enrollment to day 30, although this did not reach statistical significance (*P* = 0.49 and *P* = 0.09, respectively). There was no significant change in NGAL over time in the prerenal AKI and no AKI subgroups. Twenty-eight patients who had samples available at all 3 time points are depicted in Supplement Figure 4a (see Supplementary Digital Content 1, http://links.lww.com/CTG/A609). The trend in NGAL levels for the 110 patients with enrollment and day 5 samples available is depicted in Supplemental Figure 4b (see Supplementary Digital Content 1, http://links.lww.com/CTG/A609).

## DISCUSSION

In this US cohort, we report that urinary NGAL demonstrates strong statistical performance in differentiating ATN from other types of AKIs in cirrhosis. In addition, NGAL holds value in improving prediction of short-term mortality and AKI stage progression, particularly when used in concert with the existing models such as MELD score.

In the current practice era, there are important therapeutic implications in distinguishing HRS from ATN. Because many practitioners struggle with the clinical overlap between the 2 syndromes, an objective diagnostic test has the ability to improve the speed and accuracy of diagnosis when used in conjunction with guideline-driven approaches to AKI in cirrhosis (3,9). Prompt diagnosis of HRS allows for earlier initiation of vasoconstrictors, which has been associated with improved rate of HRS reversal in multiple studies (31–36). Furthermore, better diagnostic accuracy will limit vasoconstrictor exposure and excessive and potentially deleterious albumin dosing in those unlikely to respond to improved renal perfusion, such as patients with ATN. HRS-directed vasoconstrictors such as terlipressin and norepinephrine have meaningful side effect profiles, including ischemia and development of respiratory compromise (16,17), and thus should be limited to those with HRS who are most likely to benefit from therapy. Our median enrollment time of 3 days from admission allows for 48 hours of albumin resuscitation as per guidelines—a volume challenge that helps to eliminate prerenal AKI from the differential diagnosis of AKI in cirrhosis. At that point, a low NGAL level has an 85% negative predictive value to exclude ATN. Although these findings should be validated at other sites, we believe our case mix is representative of the general inpatient population of cirrhosis and AKI, which therefore makes NGAL a meaningful addition to the diagnostic armamentarium.

Our results build on similar contributions on this topic in the literature. Other groups have shown that NGAL performs well in distinguishing the type of AKI in cirrhosis (21–23). The largest and most rigorous analysis of urinary NGAL in this population was published by Huelin et al. (21) in 2019. This cohort examined 320 cases of AKI in cirrhosis from 214 unique patients who were hospitalized at a single-liver transplant center in Barcelona, Spain. We believe these data to be representative of the European practice experience; thus, we replicated several of their analyses to compare our North American results with theirs. We validated several of their findings, including (i) a NGAL's accuracy in differentiating ATN from other AKIs (C statistic 0.87 in Barcelona compared with 0.77 in our cohort), (ii) a similar optimal cutpoint (220 versus 243 μg/g), and (iii) that NGAL served as a strong short-term predictor of mortality. The Barcelona population was demographically similar to ours and used the same NGAL assay as in our study. However, there are several key differences in practice patterns between sites, including Barcelona's dedicated liver unit (whereas the hepatology service serves as a consultant to medicine teams at our hospital), lack of availability of terlipressin in the United States, and different availability/triage processes for liver transplantation. In addition, Huelin et al. had a more stringent definition of ATN, requiring a high urine sodium excretion and/or a high urine osmolality. Because ATN is a clinical diagnosis that does not include guideline parameters for urine studies (37), we did not perform these assays in our study. Despite these facts, we came to largely the same conclusions, which supports that NGAL may be useful across a variety of practice environments. We should highlight that our NGAL cutoffs differ from those seen in meta-analyses of studies examining NGAL to predict severe AKI (38), although this is not surprising given our cirrhotic population is not well represented in these studies and we were measuring NGAL in extant AKI rather than trying to predict a rise in creatinine.

It is worth considering the function of urinary NGAL through the lens of the pathophysiology of HRS. Ultimately, any diagnostic test for HRS is performed in service of identifying better candidates for vasoconstrictor therapy. Improvement in kidney function in HRS depends on 3 things: (i) improved effective circulating volume and increased renal blood flow, (ii) preserved microvascular circulation such that increased renal perfusion can be properly perceived by the nephron's filtering units, and (iii) sufficiently intact renal tubules that can respond to improved perfusion. Urinary NGAL addresses the third component of this process. Low NGAL suggests relatively preserved renal parenchyma, highlighting the structure-function disconnect inherent in HRS. Although renal blood flow is difficult to measure noninvasively, multiple groups have suggested an increase in mean arterial pressure of 10–15 mm Hg as a surrogate to guide vasoconstrictor dosing (39–41). The remaining component—microvascular circulation—is arguably the hardest to quantify, given the complex pathophysiologic relationships between systemic inflammatory response seen in decompensated cirrhosis (42–47), the need for preserved endothelial cell function (24), and other potential renal-specific factors that are not captured by available testing. We would encourage further exploration of microvascular inflammation in advanced liver disease because it holds potential for discovery of new targets of therapy.

NGAL was also an independent predictor of short-term mortality, and when added to the MELD score, improved its prognostic ability. This echoes findings from a multicenter European cohort of patients with acute-on-chronic liver failure and results reported by Huelin et al*.* in their AKI cohort (21,23). There are several confounders to note here, including that worsening AKI stage was also associated with mortality and that the performance of MELD score varies from region to region (5–7,48). However, it has long been established that the mortality risk of patients with AKI in cirrhosis is underrepresented by the MELD score (49,50). In addition, NGAL is also upregulated by liver injury and infection, which may confound results (20,23,51). Reassuringly, we were able to adjust for these factors in multivariable analysis, which persistently demonstrated a stable statistical signal. In addition, it is reassuring to see the stepwise increases in NGAL between no AKI (19 μg/g), prerenal AKI (43 μg/g), HRS (110 μg/g), and ATN (337 μg/g), suggesting that urinary values are indeed reflective of parenchymal kidney injury, rather than expression because of infection or liver damage.

This article should be interpreted in the context of its limitations. Our study was performed at a single liver-transplant referral center, which limits generalizability. The incidence and outcomes of complications of cirrhosis such as AKI may vary from center to center based on local standard of care and MELD-thresholds for liver transplant. However, the replication of results from Barcelona and the apparent straightforward nature of interpreting an objective test such as NGAL suggests that the assay could be implemented in diverse environments. Many of the diagnoses in this study (including cirrhosis, HRS, and ATN) rely on clinical assessment and lack gold standards, such as biopsy tissue. The limitations of serum creatinine to diagnosis AKI in cirrhosis, for example, have been well documented (12). The same limitations of evaluating kidney biomarker performance against creatinine-based definitions of AKI from the general nephrology literature should be noted here (52). Given the complex nature of this patient population, no single test is likely to discriminate ATN from HRS; the clinical use of tools such as NGAL should be taken in the context of the larger clinical presentation. Despite these limitations, we provide important supporting data for NGAL's role in the approach to AKI in cirrhosis.

In conclusion, urinary NGAL may differentiate the type of AKI in cirrhosis and may improve prediction of short-term mortality. It holds immediate clinical potential to immediately affect management of AKI in cirrhosis and warrants further investigation in this population.

## CONFLICTS OF INTEREST

**Guarantor of the article:** Andrew S. Allegretti.

**Specific author contributions:** A.S.A.: drafted the article. A.S.A., S.K., S.U.N., R.T.C.: involved in study design. X.V.P., P.E., S.A.S., S.K., J.G.F., A.N.: involved in sample collection/processing. S.Z.: performed statistical analysis. D.A.S., L.A.J., N.K., H.M.W., K.R.R., J.M.B., M.K.N., G.G.T., J.C.Q.V.: involved in analysis and interpretation of results. All authors read and approved the final version of the article.

**Financial support:** A.S.A. was supported by American Heart Association Award 18CDA34110131.

**Potential competing interests:** A.S.A., H.M.W., and K.R.R. have served on scientific advisory boards for Mallinckrodt Pharmaceuticals. R.T.C. received institutional grant support from Synlogic and Kaleido. J.M.B. has served on scientific advisory boards for Mallinckrodt and consulted for Chiasma. X.V.P. has served on scientific advisory boards for Astra Zeneca. J.C.Q.V. has served on scientific advisory boards for Mallinckrodt Pharmaceuticals and Travere Therapeutics, has served on the speaker bureau for Otsuka Pharmaceuticals, and has consulted for Bayer.

**Study Highlights**

**WHAT IS KNOWN**

✓ Similar to data out of Europe, urinary neutrophil gelatinase-associated lipocalin (NGAL) can accurately differentiate acute tubular necrosis from other types of acute kidney injuries (AKIs) in cirrhosis.

**WHAT IS NEW HERE**

✓ Urinary NGAL may serve as a short-term predictor of mortality in cirrhosis and AKI beyond Model for End-Stage Liver Disease score.

**TRANSLATIONAL IMPACT**

✓ Urinary NGAL is a useful diagnostic and prognostic tool in AKI and cirrhosis.

## Supplementary Material

SUPPLEMENTARY MATERIAL
