# Supplementary material for: Urinary NGAL as a Diagnostic and Prognostic Marker for Acute Kidney Injury in Cirrhosis: A Prospective Study
Source: Clin Transl Gastroenterol. 2021 May 11;12(5):e00359. doi: 10.14309/ctg.0000000000000359 (PMC8116001; doi:10.14309/ctg.0000000000000359)

**Supplement** **Figure 1**: Ninety-day probability of survival by urinary NGAL (mcg/g creatinine). Patients were divided by NGAL tertile at study enrollment (P <0.001).

**
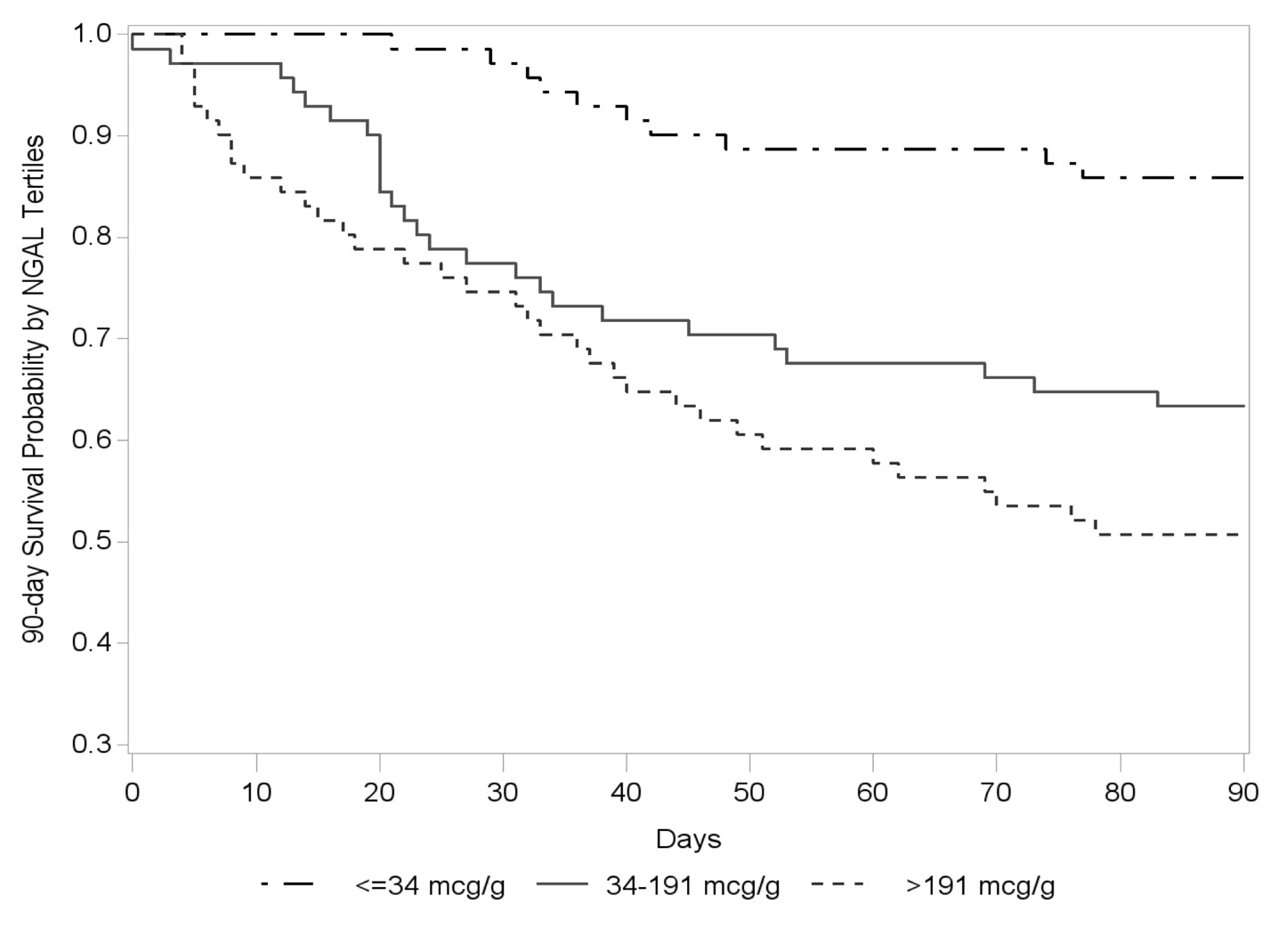
**

**Supplement** **Figure 2**: Forest plots of urinary NGAL’s hazard ratios for 90-day mortality

Key: MELD (Model for End Stage Liver Disease), CLIF-C ACLF (Chronic Liver Failure Consortium Acute-on-Chronic Liver Failure Score, AKI (acute kidney injury). Hazard ratios are presented with 95% confidence intervals. Model 1: adjusted for age and MELD score. Model 2: adjusted for age, MELD score, and presence of infection. Model 3: excluding 18 subjects with hepatocellular carcinoma.

**Supplement Figure 3**: Added predictive value of NGAL for 90-day transplant-free survival and 90-day survival to prognostic models in cirrhosis, by type of acute kidney injury subgroup

**Table 3a**: No Acute Kidney Injury (n = 52)

|  | NGAL |  | MELD | MELD + NGAL | P value |  | MELD-Na | MELD-Na + NGAL | P-value |  | CLIF-C ACLF | CLIF-C ACLF + NGAL | P value |
| --- | --- | --- | --- | --- | --- | --- | --- | --- | --- | --- | --- | --- | --- |
| Transplant-free survival |  |  |  |  |  |  |  |  |  |  |  |  |  |
| C statistic | 0.418 |  | 0.581 | 0.601 | 0.393 |  | 0.637 | 0.657 | 0.381 |  | 0.701 | 0.708 | 0.289 |
| Category-free NRI |  |  |  | -0.476 | 0.275 |  |  | -0.095 | 0.827 |  |  | -0.167 | 0.702 |
| IDI |  |  |  | 0.001 | 0.799 |  |  | 0.001 | 0.828 |  |  | 0.001 | 0.606 |
|  |  |  |  |  |  |  |  |  |  |  |  |  |  |
| Overall survival |  |  |  |  |  |  |  |  |  |  |  |  |  |
| C statistic | 0.418 |  | 0.581 | 0.601 | 0.393 |  | 0.637 | 0.657 | 0.381 |  | 0.701 | 0.708 | 0.289 |
| Category-free NRI |  |  |  | -0.476 | 0.275 |  |  | -0.095 | 0.827 |  |  | -0.167 | 0.702 |
| IDI |  |  |  | 0.001 | 0.799 |  |  | 0.001 | 0.828 |  |  | 0.001 | 0.606 |

**Table 3b**: Prerenal Acute Kidney Injury (n = 57)

|  | NGAL |  | MELD | MELD + NGAL | P value |  | MELD-Na | MELD-Na + NGAL | P-value |  | CLIF-C ACLF | CLIF-C ACLF + NGAL | P value |
| --- | --- | --- | --- | --- | --- | --- | --- | --- | --- | --- | --- | --- | --- |
| Transplant-free survival |  |  |  |  |  |  |  |  |  |  |  |  |  |
| C statistic | 0.617 |  | 0.692 | 0.712 | 0.472 |  | 0.673 | 0.705 | 0.257 |  | 0.549 | 0.617 | 0.208 |
| Category-free NRI |  |  |  | -0.061 | 0.826 |  |  | 0.121 | 0.660 |  |  | 0.226 | 0.406 |
| IDI |  |  |  | 0.045 | 0.195 |  |  | 0.047 | 0.180 |  |  | 0.070 | 0.102 |
|  |  |  |  |  |  |  |  |  |  |  |  |  |  |
| Overall survival |  |  |  |  |  |  |  |  |  |  |  |  |  |
| C statistic | 0.629 |  | 0.685 | 0.694 | 0.665 |  | 0.660 | 0.681 | 0.465 |  | 0.616 | 0.626 | 0.810 |
| Category-free NRI |  |  |  | -0.056 | 0.849 |  |  | -0.108 | 0.710 |  |  | 0.271 | 0.350 |
| IDI |  |  |  | 0.021 | 0.427 |  |  | 0.023 | 0.399 |  |  | 0.034 | 0.291 |

**Table 3c**: Hepatorenal Syndrome (n = 55)

|  | NGAL |  | MELD | MELD + NGAL | P value |  | MELD-Na | MELD-Na + NGAL | P-value |  | CLIF-C ACLF | CLIF-C ACLF + NGAL | P value |
| --- | --- | --- | --- | --- | --- | --- | --- | --- | --- | --- | --- | --- | --- |
| Transplant-free survival |  |  |  |  |  |  |  |  |  |  |  |  |  |
| C statistic | 0.594 |  | 0.599 | 0.639 | 0.400 |  | 0.617 | 0.644 | 0.590 |  | 0.569 | 0.657 | 0.153 |
| Category-free NRI |  |  |  | 0.144 | 0.627 |  |  | 0.093 | 0.754 |  |  | 0.218 | 0.463 |
| IDI |  |  |  | 0.027 | 0.127 |  |  | 0.030 | 0.104 |  |  | 0.048 | 0.040 |
|  |  |  |  |  |  |  |  |  |  |  |  |  |  |
| Overall survival |  |  |  |  |  |  |  |  |  |  |  |  |  |
| C statistic | 0.657 |  | 0.505 | 0.664 | 0.187 |  | 0.519 | 0.680 | 0.101 |  | 0.550 | 0.694 | 0.113 |
| Category-free NRI |  |  |  | 0.548 | 0.044 |  |  | 0.677 | 0.013 |  |  | 0.613 | 0.024 |
| IDI |  |  |  | 0.139 | 0.007 |  |  | 0.146 | 0.006 |  |  | 0.132 | 0.010 |

**Table 3d**: Acute Tubular Necrosis (n = 49)

|  | NGAL |  | MELD | MELD + NGAL | P value |  | MELD-Na | MELD-Na + NGAL | P-value |  | CLIF-C ACLF | CLIF-C ACLF + NGAL | P value |
| --- | --- | --- | --- | --- | --- | --- | --- | --- | --- | --- | --- | --- | --- |
| Transplant-free survival |  |  |  |  |  |  |  |  |  |  |  |  |  |
| C statistic | 0.614 |  | 0.596 | 0.628 | 0.547 |  | 0.576 | 0.614 | 0.486 |  | 0.684 | 0.696 | 0.807 |
| Category-free NRI |  |  |  | 0.428 | 0.144 |  |  | 0.428 | 0.144 |  |  | 0.523 | 0.075 |
| IDI |  |  |  | 0.037 | 0.133 |  |  | 0.037 | 0.135 |  |  | 0.050 | 0.093 |
|  |  |  |  |  |  |  |  |  |  |  |  |  |  |
| Overall survival |  |  |  |  |  |  |  |  |  |  |  |  |  |
| C statistic | 0.593 |  | 0.568 | 0.683 | 0.072 |  | 0.578 | 0.683 | 0.094 |  | 0.490 | 0.587 | 0.469 |
| Category-free NRI |  |  |  | 0.507 | 0.076 |  |  | 0.507 | 0.076 |  |  | 0.587 | 0.040 |
| IDI |  |  |  | 0.079 | 0.046 |  |  | 0.078 | 0.048 |  |  | 0.072 | 0.059 |

Key: MELD (Model for End-Stage Liver Disease), CLIF-C ACLF (Chronic Liver Failure Consortium Acute-on-Chronic Liver Failure Score), NRI (net reclassification index), IDI (integrated discrimination increment)

**Supplement Figure 4**: (Panel A – Top) Time course of median urinary NGAL (mcg/g creatinine) at study enrollment, day 5, and day 30, classified by type of acute kidney injury (AKI), among 28 subjects with samples available at all time points. (Panel B – Bottom) Time course of median urinary NGAL (mcg/g creatinine) at study enrollment and day 5, classified by type of acute kidney injury (AKI), among 110 subjects with samples available at both time points


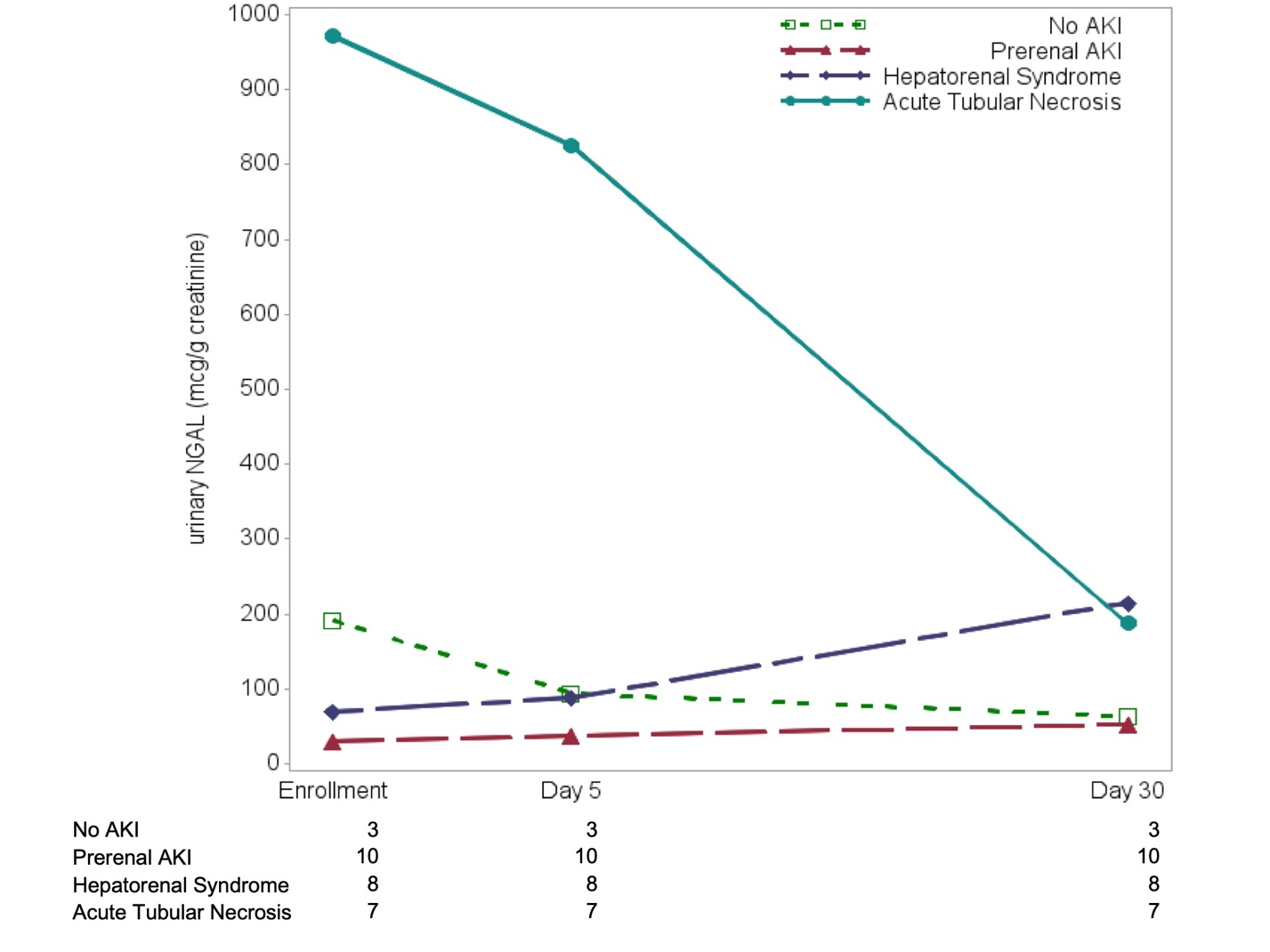


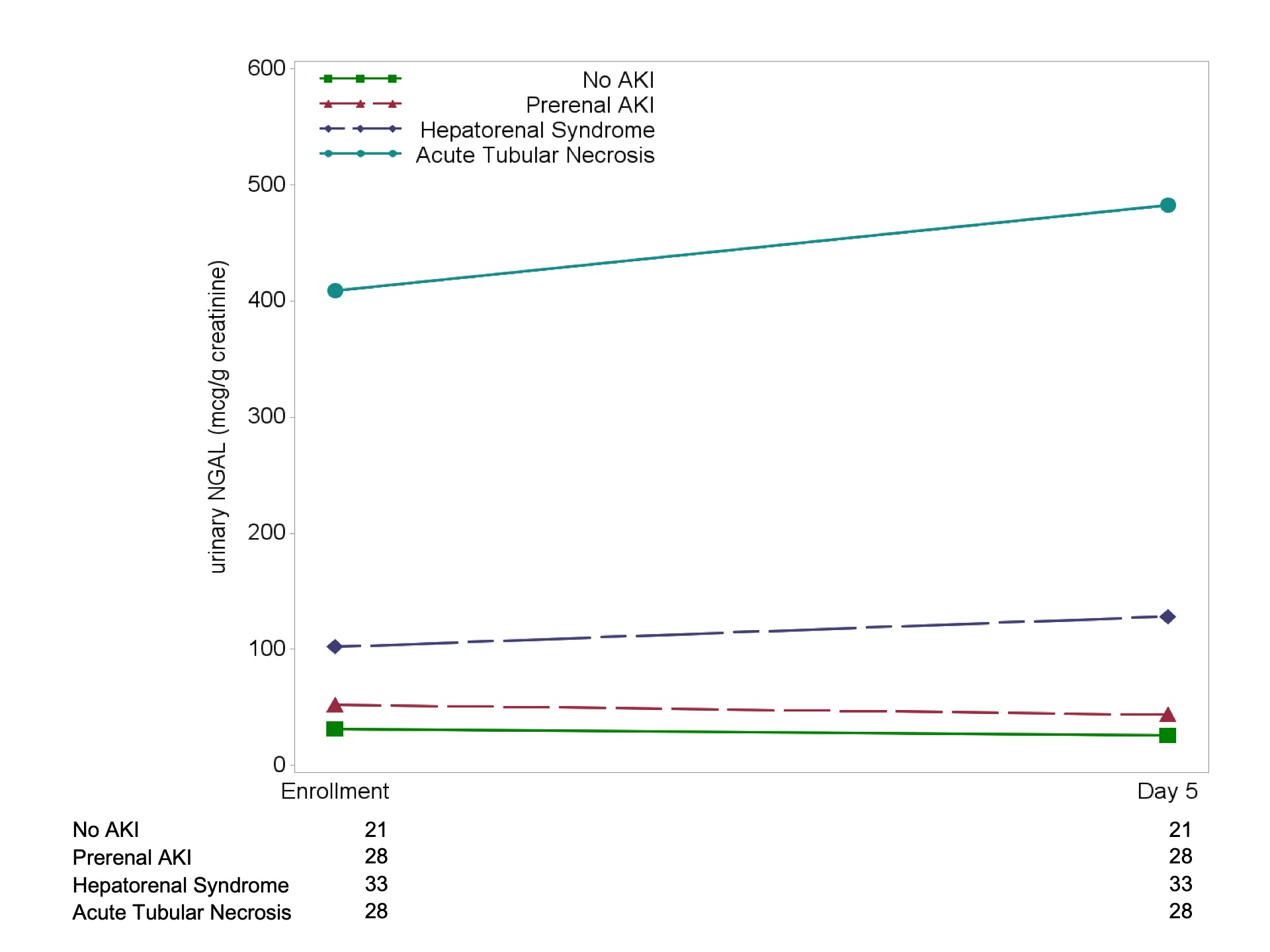

Supplement: SUPPLEMENTARY MATERIAL [file ct9-12-e00359-s001.docx]
